# Early Mortality in Paroxysmal Nocturnal Hemoglobinuria

**DOI:** 10.7759/cureus.47225

**Published:** 2023-10-17

**Authors:** Anne Lykke Sørensen, Dennis Lund Hansen, Henrik Frederiksen

**Affiliations:** 1 Department of Hematology, Odense University Hospital, Odense, DNK; 2 Department of Clinical Research, University of Southern Denmark, Odense, DNK

**Keywords:** epidemiology, cohort study, comorbidity, survival, mortality, paroxysmal nocturnal hemoglobinuria, pnh

## Abstract

Objectives: The elevated mortality risk among patients with paroxysmal nocturnal hemoglobinuria (PNH) has been suggested to derive from a high risk of thromboembolism (TE); however, the risks of coexisting cardiovascular risk factors are not well described. We studied mortality associated with PNH taking comorbidity and treatment into account.

Methods: Patients with PNH (n=115) were identified in the 1977-2016 Danish National Patient Register (DNPR). For each patient with PNH, we identified 50 age- and sex-matched general population comparators. Using the Kaplan-Meier estimator and Cox regression, we compared the overall survival of patients with comparators. Cumulative incidences were used to analyze the effects of comorbidity and the causes of death.

Results: One-year survival among patients and comparators was 92.2% and 99.4%, and after 10 years, it was 68.4% and 85.8%, respectively. Early mortality was associated with older age, higher levels of comorbidity, and solid malignancies prior to PNH diagnosis. The leading causes of death were infections and associated hematological diseases. Patients with early mortality were less likely to have received treatment with eculizumab and/or warfarin. Cardiovascular risk factors were evenly distributed between patients and comparators at diagnosis.

Conclusion: We conclude that early mortality in PNH is associated with older age, cardiovascular comorbidity, and hematological malignancies.

## Introduction

Paroxysmal nocturnal hemoglobinuria (PNH) is an acquired clonal hematopoietic stem cell disease causing non-immune hemolysis with an incidence rate of 0.35 cases per 100,000 people per year and an overall prevalence rate of 3.81 per 100,000 [1].

Manifestations of PNH are caused by a mutation in the phosphatidylinositol glycan class A and possibly in the class T gene (PIG-A and PIG-T) [2]. In healthy individuals, this gene is responsible for the synthesis of glycosylphosphatidylinositol (GPI) anchors used to attach surface proteins to red blood cells (RBCs). A deficiency of GPI anchors blocks the RBCs from attaching CD55 and CD59, two essential enzymes in preventing random complement attack [2]. Thus, without this protection, RBCs are susceptible to complement attacks causing intravascular hemolysis [3].

Patients with PNH can present with a wide range of disease manifestations from fully compensated asymptomatic hemolysis to severe anemia with reduced quality of life from fatigue, thromboembolism (TE), abdominal pain, smooth muscle dystonia, and death [4]. Prior to the introduction of complement-inhibiting agents such as eculizumab, treatment options included supportive care, anticoagulation, and hematopoietic stem cell transplantation [5,6]. With these treatment options, the median survival of PNH patients ranged from 10 to 30 years [7,8]. However, from the pivotal studies of eculizumab including 195 patients, it was shown that complications and prognosis improved and the reported risk of TE was reduced from 32.6% to 3.6%, resulting in a three-year survival proportion of 97.6% [6]. Similarly, patients with PNH treated with eculizumab had a reported four- to five-year survival of 95.5%-98.3% compared with 66.8%-79.7% in untreated patients [9].

Death in PNH is often attributed to TE. A study from 1995 of 48 patients (for whom the cause of death was known) illustrated that TE was a contributing cause of death in 28 patients [7]. Although TE is a well-recognized complication in PNH, it is unknown how other cardiovascular risk factors such as diabetes and hypertension affect mortality [4,10]. Using a nationwide matched cohort design including PNH patients and age- and sex-matched comparators from the general population, we investigated the effect of comorbidities and cardiovascular risk factors on survival among patients with PNH in Denmark during 39 years of observation.

## Materials and methods

Data sources and patients

Denmark provides tax-financed universal national healthcare, and the study population derives from the entire Danish population through routinely registered national health data. The unique and permanent civil registration number (CRN) allows individual-level linkage between all Danish registries [11,12]. For this study, we linked data from the Danish National Patient Register (DNPR), the Danish National Prescription Registry, and the Danish Register of Causes of Death [13,14]. The DNPR comprises all inpatient hospital contacts since 1977 and associated diagnoses using the International Classification of Diseases (ICD). Since 1994, hospital outpatient specialist clinic visits and emergency room contacts have also been included using the ICD-10, whereas ICD-8 was used before 1994. Private hospitals are included in the DNPR, but none of them are engaged in the management of hematological disorders or associated complications [12,15]. The diagnosis codes of cardiovascular disorders, hematological cancers, and hemolytic disorders within this registry have previously been validated, all with high positive predictive values [13,16]. The DNPR contains limited information regarding hospital treatments, but the registration is incomplete.

The present study derives from the Danish Hemolysis Cohort, a cohort comprising all patients with hemolytic disorders identified from the above-described registries [13,14,17,18]. In the Danish Hemolysis Cohort, we identified all patients diagnosed with PNH in Denmark between January 1, 1977, and December 31, 2016. To increase the reliability of the diagnosis, we excluded patients only registered with a PNH diagnosis from surgical departments or when PNH was only registered as a post-mortem diagnosis [13].

We used the DNPR to identify hospital-registered comorbidity at baseline such as cardiovascular comorbidity, cardiovascular risk factors, and associated hematological disorders (e.g., aplastic anemia (AA) or myelodysplastic syndrome (MDS)). A detailed list of the diagnosis codes is shown in the Appendices. Identification of comorbidities and risk factors was enhanced by the individual-level data on prescription drugs available from the Danish National Prescription Registry since 1994 [19]. We used the Anatomical Therapeutic Chemical (ATC) codes to identify drugs associated with specific comorbidities, e.g., insulin to identify diabetes or digoxin to identify cardiac arrhythmia (Appendices).

The Danish Register of Causes of Death holds information on immediate and underlying causes of death registered by the doctors who issued a death certificate in Denmark. This register is updated annually, and we included causes of death from 1977 to 2016 [20]. We used the recommended main underlying cause of death to assess cause-specific mortality among patients with PNH and comparators.

Comparators and follow-up

**Figure 1:**
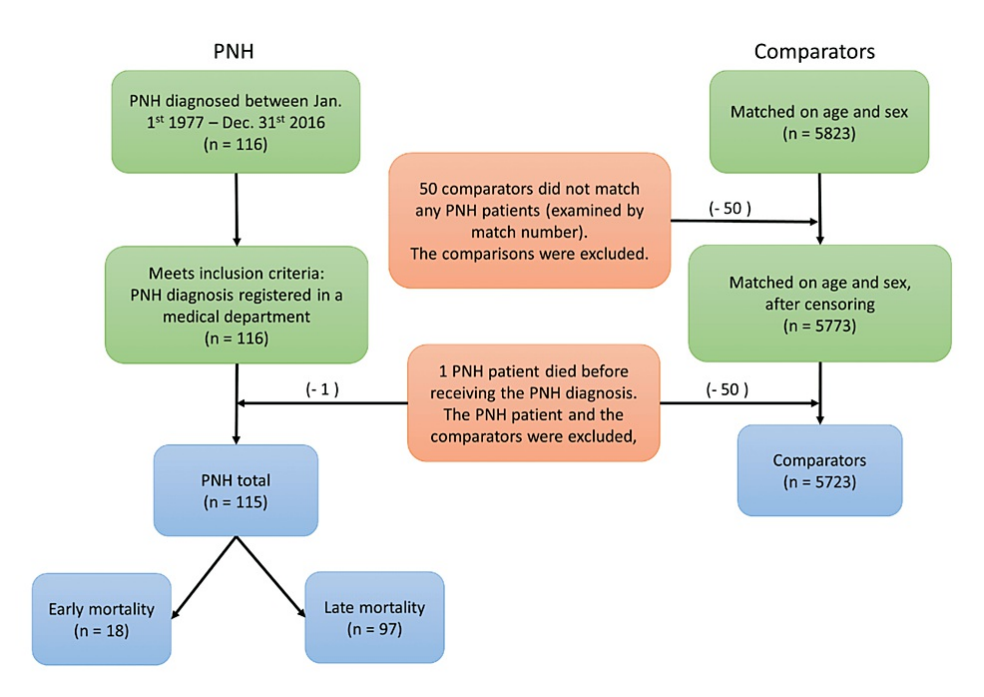
Flowchart of cohorts During the analysis, it was found that one PNH patient did not have any registered cause of death. This person and the associated comparators were excluded (red box). Also, 50 comparators were excluded because no matched PNH patient was found (red box). The PNH cohort was subdivided into two groups: short survival (PNH patients who died within two years of diagnosis) and long survival (PNH patients who lived longer). The blue boxes illustrate the final groups. Left: PNH cohort, right: comparators PNH: paroxysmal nocturnal hemoglobinuria

For each patient with PNH, we identified up to 50 age- and sex-matched comparators from the general population without PNH, each allotted an index date corresponding to the date of diagnosis of the index PNH patient (Figure 1). Information regarding comorbidity, prescription drugs, causes of death, etc. were obtained for both patients and comparators. Patients and comparators were followed from inclusion to the first of emigration, death, or end observation time on December 31, 2016.

Mortality and causes of death were assessed for early versus late mortality (<2 years versus ≥2 years) after the index date.

Statistics

Proportions with 95% confidence intervals (CIs) and medians with interquartile range (IQR) were used to assess age, sex, comorbidity (such as AA, MDS, and other hematological malignancies), venous TE, cardiovascular comorbidity and risk factors, diabetes, and the use of estrogens.

The primary endpoints were overall mortality and cause-specific mortality. To describe factors associated with early mortality, we described patients who died within two years from diagnosis and patients who lived longer, separately. Overall survival was assessed using the Kaplan-Meier estimator, and the difference in survival was tested using a log-rank test. Cause of death was estimated under competing risk with non-parametric cumulative mortality proportions [21-23]. Causes of death were grouped into six main categories based on previous studies and conditions associated with PNH: TE, hemorrhage, infection, cardiovascular disease, associated hematological disease, and other causes of death [4,7,24]. Differences in survival between groups were assessed with Cox proportional hazard regression estimating unadjusted and adjusted hazard ratios. Hazard ratios were adjusted for date of diagnosis, sex, age, cardiovascular comorbidity and risk factors, diabetes, use of estrogens, preexisting hematological disease, and TE. Model assumptions were assured with Schöenfeld residuals and comparing the Kaplan-Meier curve with the univariate predicted curves. Goodness of fit was assessed with Nelson-Aalen/Cox-Snell residuals.

The statistical software package Stata (StataCorp, College Station, TX) was used for data management and analyses [25].

Ethics and data sharing

This study was approved by the Region of Southern Denmark (case number 17/10885). Registry-based research without direct contact with patients is exempt from Ethics Committee approval in Denmark.

Danish law does not allow sharing of patient data publicly. For legal reasons, individual-level patient data therefore cannot be shared, and table output containing information from less than three persons must be anonymized. The reporting of results was guided using the REporting of studies Conducted using Observational Routinely-collected health Data (RECORD) statement [26].

## Results

Overview

During the study period (1977-2016), the Danish population increased from 5.1 to 5.7 million persons. In this period, 115 patients were registered with PNH. We identified 5,723 age- and sex-matched comparators from the general population, giving a mean match rate of 49.8:1. The patients and comparators contributed 1,364 and 85,080 person-years, respectively. At diagnosis, the patients with PNH had a median age of 48.2 (IQR: 44.5-51.9), and 50.4% were females (Table 1).

**Table 1:** Basic description of the 115 patients with PNH and 5,723 age- and sex-matched general population comparators Results marked with CI are estimated as proportions presented with 95% confidence interval. Results marked with IQR are estimated as medians presented with 75th and 25th percentiles. Due to current rules on the protection of patient anonymity from Statistics Denmark, all columns with less than three persons must be anonymized. *: Cardiovascular comorbidity (atrial fibrillation and flutter, rheumatic and hypertensive heart disease, heart attack and failure, cardiomyopathy, angina pectoris, atherosclerosis) **: Cardiovascular risk factors (hyperlipidemia, hypertension, and obesity) ***: Estimated among included women PNH: paroxysmal nocturnal hemoglobinuria, AA: aplastic anemia, MDS: myelodysplastic syndrome, TE: thromboembolism, CI: confidence interval, IQR: interquartile range

Variable	PNH (n=115)	PNH with mortality within two years (n=18)	PNH who lived for two years or longer (n=97)	Comparator (n=5,723)
Age at diagnosis, years (IQR)	48.2 (44.5-51.9)	64.2 (57.0-71.3)	45.3 (41.3-49.2)	48.2 (47.6-48.7)
Age at death, years (IQR)	67.3 (62.3-72.2)	65.1 (57.8-72.4)	68.6 (61.6-75.6)	77.7 (77.0-78.3)
Women, % (CI)	50.4 (41.0-59.9)	50.0 (26.0-74.0)	50.5 (40.2-60.8)	50.5 (49.2-51.8)
Any comorbidity, % (CI)	67.8 (58.5-76.2)	83.3 (58.6-96.4)	64.9 (54.6-74.4)	52.9 (51.6-54.2)
Comorbidities at diagnosis				
AA, % (CI)	21.7 (14.6-30.4)	16.7 (3.6-41.4)	22.7 (14.8-32.3)	<3 persons
MDS, % (CI)	6.1 (2.5-12.1)	<3 persons	5.2 (1.7-11.6)	<3 persons
Hematological malignancies, % (CI)	6.1 (2.5-12.1)	<3 persons	5.2 (1.7-11.6)	0.8 (0.6-1.0)
Solid malignancies, % (CI)	6.1 (2.5-12.1)	27.8 (9.7-53.5)	<3 persons	5.9 (5.3-6.6)
TE, % (CI)	7.0 (3.1-13.2)	<3 persons	6.2 (2.3-13.0)	1.6 (1.3-1.9)
Cardiovascular risk factors at diagnosis				
Cardiovascular comorbidity, % (CI)*	13.0 (7.5-20.6)	22.2 (6.4-47.6)	11.3 (5.8-19.4)	8.1 (7.4-8.8)
Cardiovascular risk factors, % (CI)**	20.0 (13.1-28.5)	22.2 (6.4-47.6)	19.6 (12.2-28.9)	18.6 (17.6-19.6)
Diabetes with and without chronic complications, % (CI)	6.1 (2.5-12.1)	<3 persons	6.2 (2.3-13.0)	4.1 (3.6-4.7)
Age 60+ at diagnosis, % (CI)	32.2 (23.8-41.5)	61.1 (35.7-82.7)	26.8 (18.3-36.8)	32.3 (31.1-33.6)
Estrogen-based contraceptives, % (CI)***	25.9 (15.3-39.0)	<3 persons	28.6 (16.6-43.3)	26.4 (24.8-28.0)
Post-menopausal hormone replacement therapy, % (CI)***	20.7 (11.2-33.4)	<3 persons	22.4 (11.8-36.6)	13.4 (12.2-14.7)

With notable exceptions, the prevalence of morbidities and cardiovascular risk factors at diagnosis such as hypertension, diabetes, and obesity was generally balanced between patients with PNH and the comparators (Table 1). At inclusion, 7% of patients and 1.6% of comparators were registered with a prior or coincident venous TE event (Table 1). Concurrent AA or MDS was recorded almost exclusively among patients with PNH, where 21.7% had AA and 6.1% had MDS (Table 1).

**Figure 2:**
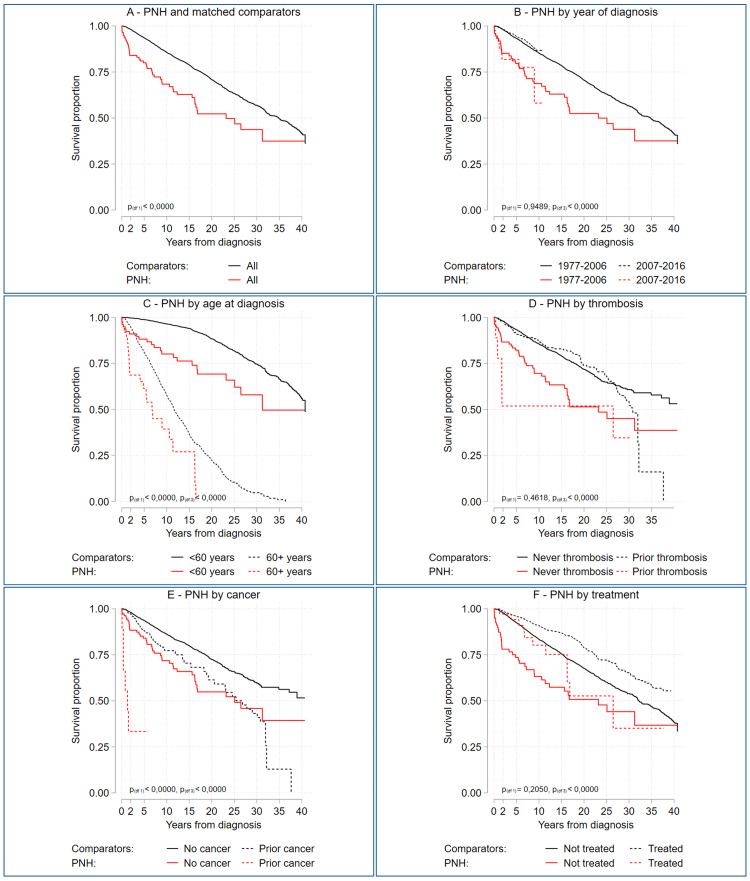
Survival among patients with PNH (A) Overall survival for patients with PNH diagnosed from 1977 to 2016 and age- and sex-matched comparators from the general population. (B) Overall survival for PNH and comparators, subdivided by year of diagnosis, with eculizumab being released in 2007. (C) Subdivided by age at diagnosis of PNH, with the older age groups starting at 60 years. (D) Subdivided by thrombosis prior to PNH diagnosis. (E) Subdivided by diagnosis of cancer prior to diagnosis of PNH. (F) Subdivided based on exposure to eculizumab and/or warfarin during follow-up. p-values in the individual graphs are given with one or, when applicable, three degrees of freedom. p-values with one degree of freedom are in B to F testing the two subgroups of PNH, whereas three degrees of freedom p-values test all four groups. PNH: paroxysmal nocturnal hemoglobinuria

Of the 115 patients with PNH, 18 died within two years of diagnosis. When comparing these patients with patients who survived longer than two years, comorbidities at diagnosis were more prevalent among those with early mortality, at 83.3% (95% CI: 58.6-96.4) versus 64.9% (95% CI: 54.6-74.4). Furthermore, early mortality was associated with older age (age at diagnosis: 64.2 (IQR: 57.0-71.3) versus 45.3 (IQR: 41.3-49.2)), previous solid cancer, and cardiovascular comorbidity (Table 1 and Figure 2C, 2E).

Survival, causes of death, and treatment

Mortality was highest among patients with PNH. The median age at death for patients with PNH was 67.3 years (IQR: 62.3-72.2), ten years lower than the median age at death among comparators (77.7 years (IQR: 77.0-78.3)) (Table 1). After the first year, overall survival was 92.2% (95% CI) in patients with PNH and 99.4% (95% CI) among comparators. After 10 years, 68.4% (95% CI) of patients with PNH were still alive versus 85.8% (95% CI) of the comparators (Figure 2A).

The adjusted hazard ratio (HR) for death in patients with PNH was 10.5-fold (95% CI: 6.3-17.3) the risk among comparators.

Mortality in patients was particularly elevated during the first two years after diagnosis but approached that of the general population gradually thereafter (Figure 2A). We did not observe the difference in survival for patients with PNH diagnosed before and after 2006 when eculizumab became available (Figure 2B). However, only 11 patients were registered with exposure to eculizumab either as monotherapy or in combination with warfarin (Table 2). The limited number of eculizumab-exposed patients did not allow for a specific analysis of the effects of eculizumab treatment on survival.

**Table 2:** Cumulative causes of death and treatment in 115 patients with PNH and 5,723 age- and sex-matched general population comparators Results marked with CI are estimated as proportions presented with 95% confidence interval. Due to current rules on the protection of patient anonymity from the Danish Statistic, all cells with one or two persons must be anonymized as “<3.” PNH: paroxysmal nocturnal hemoglobinuria, BMT: bone marrow transplant, TE: thromboembolism, AA: aplastic anemia, CI: confidence interval

Variable	PNH (n=115)	PNH with mortality within two years (n=18)	PNH who lived for two years or longer (n=97)	Comparator (n=5,723)
Treatment from the time of diagnosis				
BMT (CI)	2.6 (0.5-7.4)	<3 persons	<3 persons	0.0 (0.0-0.1)
-Warfarin/-eculizumab (CI)	68.7 (59.3-77.0)	94.4 (72.7-99.9)	63.9 (53.5-73.4)	95.2 (94.6-95.7)
-Warfarin/+eculizumab (CI)	5.2 (1.9-11.0)	0.0 (0.0-18.5)	6.2 (2.3-13.0)	0.0 (0.0-0.1)
+Warfarin/-eculizumab (CI)	21.7 (14.6-30.4)	<3 persons	24.7 (16.5-34.5)	4.8 (4.3-5.4)
+Warfarin/+eculizumab (CI)	4.4 (1.4-9.9)	0.0 (0.0-18.5)	5.2 (1.7-11.6)	0.0 (0.0-0.1)
Cumulative cause of death at the end of the period				
TE (CI)	0 (0.0-3.2)	0 (0.0-18.5)	0 (0.0-3.7)	0.1 (0.0-0.3)
Hemorrhage (CI)	0 (0.0-3.2)	0 (0.0-18.5)	0 (0.0-3.7)	3.0 (2.2-4.2)
Infection (CI)	9.4 (3.2-19.6)	23.6 (5.9-47.9)	7.5 (1.7-19.4)	3.9 (2.8-5.2)
Cardiovascular disease (CI)	22.3 (8.9-39.5)	<3 persons	23.5 (8.5-42.7)	19.6 (17.4-22.0)
Associated hematological disease, including AA and malignancy (CI)	20.7 (11.8-31.5)	48.4 (21.6-70.9)	16.5 (7.6-28.3)	1.0 (0.6-1.5)
Other causes of death, including solid tumor (CI)	4.7 (1.5-11.0)	<3 persons	3.3 (0.6-10.3)	16.9 (14.7-19.3)

For patients with PNH, the overall most frequently registered causes of death were cardiovascular disease and associated hematological disease accounting for 22.3% (95% CI: 8.9-39.5) and 20.7% (95% CI: 11.8-31.5), respectively, of the causes of death among the deceased patients. During the first year after diagnosis, hematological disease accounted for 3.5% (95% CI: 1.2-8.2) of the mortality among patients with PNH and 0% among comparators. After 10 years, this increased to 12.3% (95% CI: 6.6-19.7) among patients and 0.2% (95% CI: 0.1-0.3) among comparators. None of the deaths among patients with PNH was attributed to TE (Table 2 and Table 3).

**Table 3:** Overall survival and cause-specific death in 115 patients with PNH and 5,723 age- and sex-matched general population comparators Results marked with CI are estimated as proportions presented with 95% confidence interval. Due to current rules on the protection of patient anonymity from the Danish Statistic, all cells with one or two persons must be anonymized as “<3.” PNH: paroxysmal nocturnal hemoglobinuria, TE: thromboembolism, AA: aplastic anemia, CI: confidence interval

Variable	End of first year		End of third year		End of fifth year		End of 10th year	
	PNH	Comparator	PNH	Comparator	PNH	Comparator	PNH	Comparator
Overall survival (CI)	92.2 (85.5-95.8)	99.4 (99.2-99.6)	84.0 (75.8-89.6)	96.6 (96.1-97.1)	80.0 (71.2-86.4)	93.5 (92.8-94.2)	68.4 (58.1-76.7)	85.7 (84.6-86.7)
Cause-specific death								
TE (CI)	0.0	0.0	0.0	0.0	0.0	0.0 (0.0-0.1)	0.0	0.1 (0.0-0.2)
Hemorrhage (CI)	0.0	0.0	0.0	0.1 (0.1-0.3)	0.0	0.3 (0.2-0.4)	0.0	0.5 (0.3-0.7)
Infection (CI)	0.0	0.0	2.8 (0.8-7.3)	0.1 (0.1-0.3)	2.8 (0.8-7.3)	0.2 (0.1-0.4)	4.0 (1.3-9.3)	0.6 (0.4-0.9)
Cardiovascular disease (CI)	<3 persons	0.2 (0.1-0.4)	<3 persons	1.3 (1.0-1.6)	2.9 (0.8-7.5)	2.3 (1.9-2.7)	4.0 (1.3-9.1)	5.4 (4.7-6.0)
Associated hematological disease, including AA and malignancy (CI)	3.5 (1.2-8.2)	0.0	6.3 (2.8-11.9)	0.0	7.4 (3.4-13.3)	0.0 (0.0-0.1)	12.3 (6.6-19.7)	0.2 (0.1-0.3)
Other causes of death, including solid tumor (CI)	<3 persons	0.2 (0.1-0.3)	<3 persons	0.9 (0.7-1.2)	<3 persons	1.8 (1.5-2.2)	3.1 (0.8-8.2)	3.8 (3.2-4.3)

In patients with PNH who died within two years from diagnosis, associated hematological disease (48.4% (95% CI: 21.6-70:9)) or infections (23.6% (95% CI: 5.9-47.9)) were the most frequent cause of death (Table 2). Among patients and comparators with late mortality, cardiovascular disease was an equally frequently registered cause of death, whereas death from associated hematological diseases was significantly more frequent among patients (16.5% (95% CI: 7.6-28.3)) than comparators (1% (95% CI: 0.6-1.5)) (Table 2). Furthermore, patients with early mortality after PNH diagnosis were often untreated with warfarin or eculizumab (94.4% (95% CI: 72.7-99.9)) compared to patients who lived longer (63.9% (95% CI: 53.5-73.4)) (Table 2, Figure 2F). The same trend is suggested from the Kaplan-Meier plot comparing patients with PNH who received treatment (eculizumab and/or warfarin) to the patients with PNH who did not receive any such treatment (Figure 2F). However, a post hoc analysis indicated that the difference in survival between treated and untreated patients with PNH was only significant within the first five years following diagnosis (p=0.01 at five years, p=0.10 at 10 years).

Only a minority of the patients with PNH were treated with eculizumab (5.2% (95% CI: 1.9 -11.0)), and a further 4.4% (95% CI: 1.4-9.9) received both eculizumab and warfarin. Hematopoietic stem cell transplant was undertaken in less than three patients with PNH.

## Discussion

Using a nationwide cohort with long and complete follow-up, we confirm that mortality is higher among patients with PNH than in the general population. Mortality is particularly increased during the first two years after PNH diagnosis and was associated with older age, preexisting solid cancer, and a higher prevalence of comorbidities at the time of diagnosis. Death due to associated hematological disease and infections prevailed within the first two years from diagnosis, accounting for 48.4% and 23.6% of the fatalities, respectively. Death due to cardiovascular disease was a frequent cause of death both among PNH patients and comparators, but TE was not registered as a cause of death among patients with PNH.

Our results comply with earlier notice that associated hematological diseases, including AA and malignancy, and infections are the leading causes of death in PNH [24]. However, the absence of TE as a reported cause of death is in contrast to earlier reports [7,10,24]. A French retrospective study including 460 patients with PNH, diagnosed before eculizumab became available, found that infections (24%) and Budd-Chiari syndrome (21%) were the most frequent causes of death [24]. Another study conducted before the introduction of eculizumab included 80 patients with PNH diagnosed over a period of 30 years and obtained causes of death for 48 deceased patients of whom 28 (35%) died from either venous TE or hemorrhage [7]. A study conducted from 1970 to 2013 that included 56 patients with PNH and retrospective data from medical records reported that TE episodes and cancer constituted the main causes of death, each accounting for 8.9% [27]. Taken together, these studies indicate that TE is a frequent cause of death, although we did not find this in our data. The algorithm for deriving the main cause of death in Danish registries defines the most probable main cause, and TE may well have been a contributing cause of death in our patients. Further, the main cause of death derives from the death certificate, which is susceptible to errors. This may be augmented by the decline in the autopsy rate in Denmark [20]. Together, our causes of death results should be interpreted with caution and are best suited to highlight the differences within the cohort rather than describing expected causes of death in future cohorts.

The two-year cutoff for early versus late mortality was justified by the change in slope on the survival curve for patients with PNH at two years (Figure 2A). This phenomenon was taken as an indication of a change in risk among the patients and was used to divide them into two potentially different subgroups. The distinction between early and later mortality yielded some differences in patients’ characteristics, treatment, and causes of death, but a causal relationship cannot be established. Especially as the age at death is comparable in the two groups, a possibility persists that the predominant difference between the groups could be defined by delayed diagnosis in the one group, whereby the groups represent a surveillance bias and different steps in disease progression.

Our data shows that patients with early mortality were less likely to have been treated with warfarin and eculizumab. However, this observation could reflect both undertreatment and confounding from comorbidity or advanced age leading to death before treatment was initiated. In fact, patients with early mortality were characterized by increased age, previous solid cancer, cardiovascular comorbidity, and higher prevalence of comorbidities at diagnosis. Note that older age in patients with early mortality and death a year after diagnosis could often suggest that the diagnoses of PNH in these cases were delayed.

All of these characteristics are also associated with an increased risk of thrombosis. Our study could therefore suggest, but not conclude, that these high-risk patients in particular might benefit from antithrombotic treatment.

This difference between the groups could also be due to diagnoses before eculizumab became available in 2007, when the overall mortality would be expected to be higher. Further, we collected our data over a period of 39 years, which introduces the risk of cohort effects with increased survival among both patients and comparators. Supportive care has improved over time and would be expected to increase survival; however, our data do not deliver firm evidence of this. The comparators increased survival slightly before and after 2007, but the patients with PNH have overlaying survival curves before and after (Figure 2B).

Our inclusion of comorbid conditions and cardiovascular risk factors as covariates was based on hospital diagnoses from routine registrations. The registry-based data do not include granular results from medical files such as body mass index and smoking history. This shortcoming in data may disproportionately affect comparators that are not routinely followed in the hospital and may therefore have incomplete data regarding, e.g., obesity or hypertension. Noteworthy, most of our included cardiovascular risk factors were evenly distributed among patients and comparators, indicating that this bias is unlikely to play a major role.

Our patients with PNH were captured using hospital diagnosis registrations from non-surgical departments and did not include post-mortal diagnosis. We excluded patients captured only through such registrations because our previous validation study found that a hemolytic anemia diagnosis in these settings is often not valid [13]. This study also validated the hemolysis diagnosis registered in the DNPR and found that the diagnosis of acquired hemolytic anemia (PNH included) had a positive predictive value (PPV) of 83.4% (95% CI: 77-89). PNH, as an independent diagnosis, had a PPV of 80% (95% CI: 28-88); however, this was based on only five registrations [13]. Because of inclusion criteria, our study design could potentially lead to the exclusion of the most severely ill patients presenting with a rapidly fatal outcome such as splanchnic TE, and therefore, survival may be overestimated.

Considering the rarity of the PNH condition, our study includes a relatively high number of patients diagnosed during a 39-year period. The number of patients with PNH in this period may be higher since patients with a small clone in conjunction with, e.g., AA are not completely registered. In general, AA and not a small PNH clone would often dominate the clinical course of such patients, and the effects of the incomplete inclusion of AA-PNH patients on our results are unknown.

Despite the nationwide accrual of patients with PNH, a matched comparison group, and complete follow-up, our study has limitations. We based inclusion on register data, and therefore, we lack granular laboratory data such as PNH clone size and LDH, as well as patient-specific factors such as symptoms, BMI, and smoking habits. Therefore, we cannot disentangle PNH severity and lifestyle factors that may influence prognosis.

Another inherited limitation of the register data is that we did not have the granularity to inform on the etiology of lethal infections, as only discharge diagnosis is registered in the DNPR or the Danish Register of Causes of Death. Further, the limited number of patients with PNH who died from infections did not allow for detailed analysis of the specific infections associated with mortality [28-30].

Eculizumab exposure represents a singular limitation to our study as there is no mandatory registration of this treatment. Without complete capture of eculizumab exposure, a bias in the relative estimate between the treated and untreated groups may decrease the difference, biasing the untreated group estimate toward that of the eculizumab-treated group. We therefore focused on the chronological disjunction of patients diagnosed before or after the introduction of eculizumab in Denmark, rather than registered exposure. This partitioning will also bear a risk of bias, as patients diagnosed before but surviving at least until the time of introduction may also have been exposed to eculizumab and are selected for longer survival. Overall, the very limited number of patients registered with eculizumab treatment does not allow for conclusions regarding the isolated effect of eculizumab on survival.

## Conclusions

Our study indicates that age, previous solid cancer, cardiovascular comorbidity, and comorbidity at the time of diagnosis are associated with early mortality after PNH diagnosis. In addition, mortality due to hematological disease and infections prevail as frequent causes of death among patients with early mortality.

The increased mortality in older patients could suggest that older patients remain undiagnosed for longer periods and therefore succumb to PNH-related complications shortly after diagnosis and that undertreatment is a potential risk in these high-risk patients. The rarity of the disease calls for international collaboration to investigate rare events and complications associated with PNH, particularly when subgroups such as elderly patients are considered.

## Appendices

A detailed list of diagnosis codes used in Table 1 is presented in Table 4.

**Table 4:** ICD-8 and ICD-10 codes used in Table 1 ICD: International Classification of Diseases, AA: aplastic anemia, MDS: myelodysplastic syndrome, TE: thromboembolism, PNH: paroxysmal nocturnal hemoglobinuria

Variable name	ICD-8 diagnoses	ICD-10 diagnoses
AA	2840	DD61 DD610 DD611 DD612 DD613 DD618 DD619
Any comorbidity or risk factor at diagnosis	See codes for defining risk factors: AA, MDS, hematological malignancies, solid malignancies, venous TE, cardiovascular comorbidity (heart failure, heart attack, flutter, etc.), cardiovascular risk factors (hyperlipidemia, hypertension, and obesity), diabetes with and without chronic complications, estrogen-based contraceptive, post-menopausal hormone replacement therapy, also defined as age 60+ at diagnosis	
Cardiovascular comorbidity (atrial fibrillation and flutter, rheumatic and hypertensive heart disease, heart attack and failure, cardiomyopathy, angina pectoris, atherosclerosis)	4279 4270 4271 428 7824 410 41309 41399 44389 44500 44509 44590 44599 44020 44030	DI099 DI110 DI130 DI132 DI200 DI20 DI21 DI22 DI23 DI249 DI25 DI420 DI425 DI426 DI427 DI428 DI429 DI43 DI48 DI50 DI702 DI739 DP290
Cardiovascular risk factors (hyperlipidemia, hypertension, and obesity)	27200 27201 277 400 401 402 403 404	DE65 DE66 DE67 DE68 DE780 DE781 DE782 DE783 DE784 DE785 DI10 DI11 DI12 DI13 DI14 DI15
Diabetes with and without chronic complications	24901 24902 24903 24904 24905 24908 25001 25002 25003 25004 25005 25008 24900 24906 24907 24909 25000 25006 25007 25009 7611	DE102 DE103 DE104 DE105 DE106 DE107 DE108 DE112 DE113 DE114 DE115 DE116 DE117 DE118 DE122 DE123 DE124 DE125 DE126 DE127 DE128 DE132 DE133 DE134 DE135 DE136 DE137 DE138 DE142 DE143 DE144 DE145 DE146 DE147 DE148 DE100 DE101 DE109 DE110 DE111 DE119 DE120 DE121 DE129 DE130 DE131 DE139 DE140 DE141 DE149
Hematological malignancies	20 27599	DC77 DC78 DC79 DC80 DC81 DC81 DC83 DC84 DC85 DC86 DC88 DC90 DC91 DC92 DC93 DC94 DC95 DC96
MDS		DD460 DD461 DD462 DD464 DD467 DD469
PNH	28393	DD595
Solid malignancies	140 141 142 143 144 145 146 147 148 149 150 151 152 153 154 155 156 157 158 159 160 161 162 163 164 165 166 167 168 169 170 171 172 173 174 175 176 177 178 179 180 181 182 183 184 185 186 187 188 189 190 191 192 193 194 195 196 197 198 199	DC00 DC01 DC02 DC03 DC05 DC06 DC07 DC08 DC09 DC10 DC11 DC12 DC13 DC14 DC15 DC16 DC17 DC18 DC19 DC20 DC21 DC22 DC23 DC24 DC25 DC26 DC27 DC28 DC29 DC30 DC31 DC32 DC33 DC34 DC35 DC36 DC37 DC38 DC39 DC40 DC41 DC42 DC43 DC44 DC45 DC46 DC47 DC48 DC49 DC50 DC51 DC52 DC53 DC54 DC55 DC56 DC57 DC58 DC59 DC60 DC61 DC62 DC63 DC64 DC65 DC66 DC67 DC68 DC69 DC70 DC71 DC72 DC73 DC74 DC75 DC76 DC77 DC78 DC79 DC80
Venous TE	433 434 45099 451 452 453 671	DI26 DI801 DI803 DI808 DI809 DI819 DI630 DI633 DI743 DI829 DZ867B

Table 5 shows a detailed list of diagnosis codes used in Table 2 and Table 3.

**Table 5:** ICD-8 and ICD-10 codes used in Table 2 and Table 3 ICD: International Classification of Diseases, AA: aplastic anemia, TE: thromboembolism

Variable name	ICD-8 diagnoses	ICD-10 diagnoses
Cardiovascular disease	3910 3911 3912 3930 3940 3949 3950 3959 3960 3969 3970 3980 3989 4000 4001 4002 4003 4009 4010 4019 4020 4029 4030 4033 4039 4040 4043 4049 4100 4101 4108 4109 4110 4119 4120 4129 4130 4139 4140 4149 4230 4240 4241 4249 4250 4259 4260 4261 4269 4270 4271 4272 4279 4280 4289 4290 4299 4320 4329 4330 4339 4340 4349 4350 4359 4360 4369 4370 4379 4380 4389 4400 4401 4402 4403 4409 4430 4431 4432 4438 4439 4440 4441 4443 4444 4449 4450 4459 4500 4509 4510 4511 4518 4519 4520 4529 4530 7600 7601 7602	I109 I110 I119 I120 I129 I130 I131 I132 I139 I150 I151 I152 I158 I159 I200 I201 I208 I209 I210 I211 I212 I213 I214 I219 I220 I221 I228 I229 I230 I231 I232 I233 I234 I235 I236 I238 I240 I241 I248 I249 I250 I251 I252 I253 I254 I255 I256 I258 I259 I260 I269 I270 I271 I272 I278 I279 I280 I281 I288 I289 I300 I308 I309 I310 I311 I312
Hemorrhage	2871 4300 4309 4310 4319 4410 4411 4412 4419 4420 4560 5310 5319 5329 5339 5340 5349 5350 5691 6322 8520 8539	I600 I601 I602 I603 I604 I605 I606 I607 I608 I609 I610 I611 I612 I613 I614 I615 I616 I618 I619 I620 I621 I629 I864 I868 I710 I711 I712 I713 I714 I715 I716 I718 I719 I720 I721 I722 I723 I724 I728 I729 I770 I850 I864 I868 D693 K250 K251 K252 K253 K254 K255 K256 K257 K259 K260 K261 K262 K263 K264 K265
Infection	0001 0009 0019 0020 0021 0022 0029 0030 0039 0040 0041 0042 0043 0044 0048 0049 0050 0051 0052 0058 0059 0060 0069 0070 0071 0072 0079 0080 0081 0082 0083 0088 0089 0090 0091 0092 0099 0109 0110 0120 0121 0122 0123 0129 0130 0139 0140 0150 0151 0152 0158 0159 0160 0170 0171 0172 0173 0179 0180 0181 0189 0190 0191 0192 0193 0194 0199 0200 0201 0209 0219 0229 0230 0231 0232 0239 0249 0259 0260 0261 0269 0270 0271 0279 0300 0301 0302 0303 0309 0319 0329 0330 0331 0339 0340 0341 0350 0359 0360 0361 0368 0369 0370 0379 0380 0381 0382 0388 0389 0390 0391 0399 0400 0419 0429 0439 0440 0449 0450 0451 0459 0469 0500 0501 0509 0519 0520 0530 0540 0550 0560 0570 0571 0578 0579 0600 0601 0609 0619 0620 0621 0622 0623 0624 0629 0630 0631 0632 0639 0649 0659 0660 0670 0671 0672 0673 0674 0675 0679 0680 0681 0682 0689 0700 0719 0720 0730 0739 0740 0741 0742 0749 0750 0769 0779 0780 0781 0782 0788 0789 0790 0791 0792 0793 0794 0795 0797 0798 0799 0809 0810 0811 0812 0819 0820 0821 0822 0829 0830 0831 0832 0838 0839 0840 0841 0842 0843 0844 0845 0848 0849 0850 0851 0852 0859 0860 0868 0869 0870 0871 0878 0879 0880 0881 0889 0890 0899 0900 0901 0902 0903 0904 0905 0906 0907 0909 0910 0911 0912 0913 0918 0919 0920 0929 0930 0939 0940 0941 0949 0950 0969 0970 0971 0979 0980 0981 0982 0983 0988 0990 0991 0992 0999 1000 1008 1009 1019 1020 1021 1022 1023 1024 1025 1026 1027 1028 1029 1030 1031 1032 1033 1039 1040 1049 1100 1110 1111 1112 1118 1119 1120 1139 1149 1159 1160 1161 1162 1169 1170 1171 1172 1173 1174 1178 1179 1200 1201 1202 1203 1208 1209 1210 1211 1212 1213 1214 1219 1220 1221 1228 1229 1230 1231 1232 1233 1234 1235 1236 1239 1249 1250 1251 1252 1253 1254 1255 1258 1259 1260 1261 1268 1269 1270 1271 1272 1273 1274 1275 1279 1280 1288 1289 1299 1300 1301 1302 1309 1310 1320 1330	A000 A001 A009 A010 A011 A012 A013 A014 A020 A021 A022 A028 A029 A030 A031 A032 A033 A038 A039 A040 A041 A042 A043 A044 A045 A046 A047 A048 A049 A050 A051 A052 A053 A054 A058 A059 A060 A061 A062 A063 A064 A065 A066 A068 A069 A070 A071 A072 A073 A078 A079 A080 A081 A082 A083 A084 A085 A099 A150 A151 A152 A153 A154 A155 A156 A157 A158 A159 A160 A161 A162 A163 A164
Other causes of death, including solid tumor	1400 1401 1402 1409 1410 1411 1412 1413 1419 1420 1428 1429 1430 1431 1439 1440 1449 1450 1451 1458 1459 1460 1468 1469 1470 1480 1481 1488 1489 1490 1499 1500 1510 1511 1518 1519 1520 1528 1529 1530 1531 1532 1533 1538 1539 1540 1541 1542 1550 1551 1558 1560 1561 1561 1562 1569 1570 1578 1579 1580 1589 1590 1599 1600 1601 1602 1608 1609 1610 1618 1619 1620 1621 1630 1631 1639 1700 1701 1702 1703 1704 1705 1706 1707 1708 1709 1710 1711 1712 1713 1719 1720 1721 1722 1723 1724 1725 1726 1727 1728 1729 1730 1731 1732 1733 1734 1735 1736 1737 1738 1739 1740 1740 1800 1810 1819 1820 1829 1830 1831 1839 1840 1841 1848 1849 1850 1859 1860 1869 1870 1878 1879 1880 1890 1891 1892 1899 1900 1910 1920 1921 1922 1923 1924 1925 1929 1930 1939 1940 1941 1942 1943 1944 1948 1949 1950 1951 1959 1960 1961 1962 1963 1964 1967 1968 1969 1970 1971 1972 1973 1974 1975 1976 1977 1978 1979 1980 1981 1982 1983 1984 1985 1989 1990 1991 2300 2301 2302 2303 2304 2305 2306 2307 2309 2310 2311 2312 2313 2314 2315 2319 2320 2321 2322 2330 2340 2341 2349 2349 2350 2359 2360 2361 2362 2368 2369 2370 2371 2372 2373 2374 2375 2376 2379 2380 2381 2382 2383 2384 2385 2386 2387 2389 2390 2391 2399 3770 6345 6730 6731 6739 1428 1500 1519 1529 1530 1533 1538 1541 1542 1550 1560 1570 1578 1579 1580 1600 1602 1619 1621 1740 1800 1820 1829 1830 1880 1890 1910 1950 1959 1991	C000 C001 C002 C003 C004 C005 C006 C008 C009 C019 C020 C021 C022 C023 C024 C028 C029 C030 C031 C039 C040 C041 C048 C049 C051 C052 C058 C059 C060 C061 C062 C068 C069 C079 C080 C081 C088 C089 C090 C091 C092 C098 C099 C100 C101 C102 C103 C104 C108 C109 C110 C111 C112 C113 C118 C119 C129 C130 C131 C132 C138 C139 C140 C141 C142 C148 C150 C151 C152 C153 C154 C155 C158 C159 C160 C161 C162 C163 C164 C165 C166 C168 C169 C170 C171 C172 C173 C178 C179 C180 C181 C182 C183 C184 C185 C186 C187 C188 C189 C199 C209 C210 C211 C212 C218 C220 C221 C222 C223 C224
Other hematological disease, including AA and malignancy	2000 2001 2010 2019 2020 2021 2022 2029 2030 2039 2040 2041 2049 2050 2051 2059 2060 2061 2069 2070 2071 2072 2079 2080 2089 2090 2879 2899 2821 2822 2823 2824 2825 2829 2830 2830 2839 2041 2050 2051 2090	C810 C811 C812 C813 C814 C817 C819 C820 C821 C822 C827 C829 C830 C831 C832 C833 C834 C835 C836 C837 C838 C839 C841 C842 C844 C845 C850 C851 C857 C859 C861 C864 C865 C880 C881 C882 C883 C884 C887 C889 C900 C901 C902 C903 C910 C911 C912 C913 C914 C915 C916 C917 C918 C919 C920 C921 C922 C923 C924 C925 C927 C928 C929 C930 C931 C932 C937 C939 C940 C941 C942 C943 C944 C945 C946 C947 C950 C951 C952 C957 C959 C960 C961 C962 C963 C964 C967 C969 D460 D461 D462 D463 D464 D465 D466 D467 D469
TE	4340 4349 4440 4441 4442 4443 4444 4449 4509 4530 9950 9951	G08 G089 G951 I236 I513 I676 I740 I741 I742 I743 I744 I745 I748 I803 I81 I819 I822 I823 I829 N280 O225 O873 T817

The Anatomical Therapeutic Chemical (ATC) codes used to identify drugs associated with specific comorbidities are shown in Table 6.

**Table 6:** ATC codes ATC codes: Anatomical Therapeutic Chemical codes

Variable name	ATC codes
Cardiovascular comorbidity	C01AA C01DA
Cardiovascular risk factors	C10 C02 C03A C08CA C08DB01 C09A C09B C09C C09D A08A
Diabetes with and without chronic complications	A10
Eculizumab	L04AA25
Estrogen-based oral contraceptives	G03AA G03AB
Post-menopausal hormone replacement therapy	G03C G03D G03F
Warfarin	B01AA

The procedure codes are shown in Table 7.

**Table 7:** Procedure codes BMT: bone marrow transplantation

Variable name	Procedure codes
BMT	BOQE3 BOQE4 BOQE5 BOQE6 BOQE7
Eculizumab	ML04AA25 BWHB84
Warfarin	MB01AA
